# The CB6F1 mouse is a model for studying cognition and brain morphometry with increasing age

**DOI:** 10.31491/apt.2023.03.110

**Published:** 2023-03-29

**Authors:** Devika Gandhay, Christina Pettan-Brewer, Warren Ladiges

**Affiliations:** a Department of Comparative Medicine, School of Medicine, University of Washington, Seattle, WA, USA

**Keywords:** CB6F1 mouse, cognitive impairment, aging, brain size, mouse model of brain aging

## Abstract

Cognitive impairment associated with memory loss and dysfunctional communication is a common condition in older people. Regions of the brain have been reported to decrease in size with increasing age, but the relationship with cognitive impairment is not well understood. Inbred and hybrid mouse strains can be useful models to investigate cognitive impairment and morphological changes at older ages. CB6F1 hybrid mice, a cross between C57BL/6 and Balb/c mice, were tested for learning and memory using a radial water tread maze. Old CB6F1 male mice (30 months of age) had severe cognitive impairment, while it was virtually absent in young (6 months old) male mice. In these same mice, there was a significant decrease in sagittal flat surface area of the hippocampus and pons in old versus young animals. The aging CB6F1 mouse would be a potential model to study the relationship between changes in brain morphometry and cognitive impairment and the identification of possible therapeutic targets.

The decline of cognitive function is measured as changes in how quickly one processes information, makes a decision, and the ability to recall working memory [1]. The hippocampus is responsible for memory and learning and is located within the temporal lobe as an extension of the cerebral cortex [2]. When neuron damage occurs, such as in dementia, the hippocampus is one of the most severely affected regions of the brain [2]. In addition, the pons is responsible for receiving sensory stimuli, analysis, and motor control. Specifically, the pons acts as a pathway for nerve fibers that join the cerebellum and the cerebral cortex of the brain and is located above the medulla oblongata as part of the brain stem [3]. Shrinkage in the volume of the brain tends to occur primarily in the frontal cortex with increasing age and has been suggested to be due to a decrease in neuronal or cortical volume [4]. White matter lesions can also cause a decrease in the mass of brain regions. Shrinkage that comes with the natural course of aging can lead to cognitive decline, especially in terms of episodic memory function [5].

C57BL/6 mice crossed with Balb/c mice are designated as CB6F1 mice. They are readily available from the National Institute on Aging Aged Rodent Colony and have a more heterogeneous genetic background compared to the parenteral inbred strains. Cognitive impairment generally increases with increasing age but Has not been well documented in CB6F1 mice. To compare cognitive function in young CB6F1 mice and old CB6F1 mice a radial water tread maze [6] was used to test 23 6-month-old mice, which we characterized as “young” and 21 30-month-old mice, which we characterized as “old”. The radial water tread maze is a test for learning and memory and consists of a 30-inch circular galvanized metal enclosure with nine holes in the sides at regular intervals each with a visual image and object. An inch of room-temperature water was added to the tank and a bright light was placed overhead. Mice were placed in the center of the tank and required to find the escape hole that led to a dark safe box. Test results show that older CB6F1 mice had slower escape times than younger CB6F1 mice (Figure 1).

To gain preliminary insight into which morphological brain regions might be affected by aging and possible association with cognitive impairment, five mice from each age group were randomly selected, euthanized, and brains collected and fixed in formalin for 48 hours and then transferred to PBS. Sagittal brain sections were stained with Hematoxylin and Eosin and brain regions were outlined and labeled. Image J Software was utilized to analyze the area of each brain region measured in pixels, which was standardized to tibial length to accurately compare surface area in young and old mice. As can be seen in Figure 2, younger mice had larger hippocampal and pons areas compared to the same areas in the older mice. Figure 3 is an example of a sagittal view of the brain from one young mouse and one old mouse showing the different brain regions.

This brief report shows that cognitive impairment is prevalent in 30-month-old CB6F1 male mice, while virtually absent in 6-month-old male mice. Preliminary evidence also suggests that regions of the brain including the hippocampus and pons differ in morphometry from young to old. These observations are consistent with previous studies concluding that brain tissue undergoes structural changes due to the advancement of age in areas such as the hippocampus [7], and suggest that the aging CB6F1 mouse is a potential model to study the relationship between changes in brain morphometry and cognitive impairment. Cellular and molecular mediators could be determined, using RNA sequencing, digital imaging, and nanoscale technology, to identify possible therapeutic targets.

## Figures and Tables

**Figure 1.**
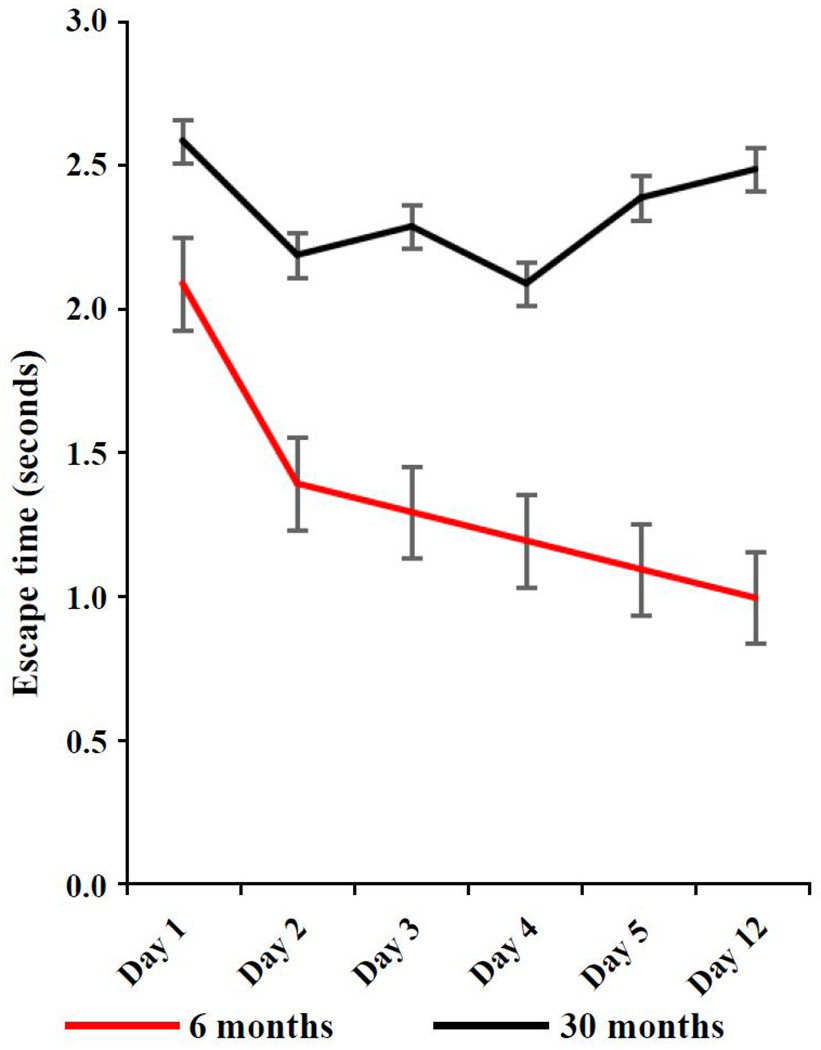
The radial water tread maze was used to assess learning and memory in young (6 months) and old (30 months) CB6F1 male mice. Young mice demonstrated the ability to learn and retain memory while old mice showed significant learning and memory impairment. N = 21-23 mice per age group.

**Figure 2.**
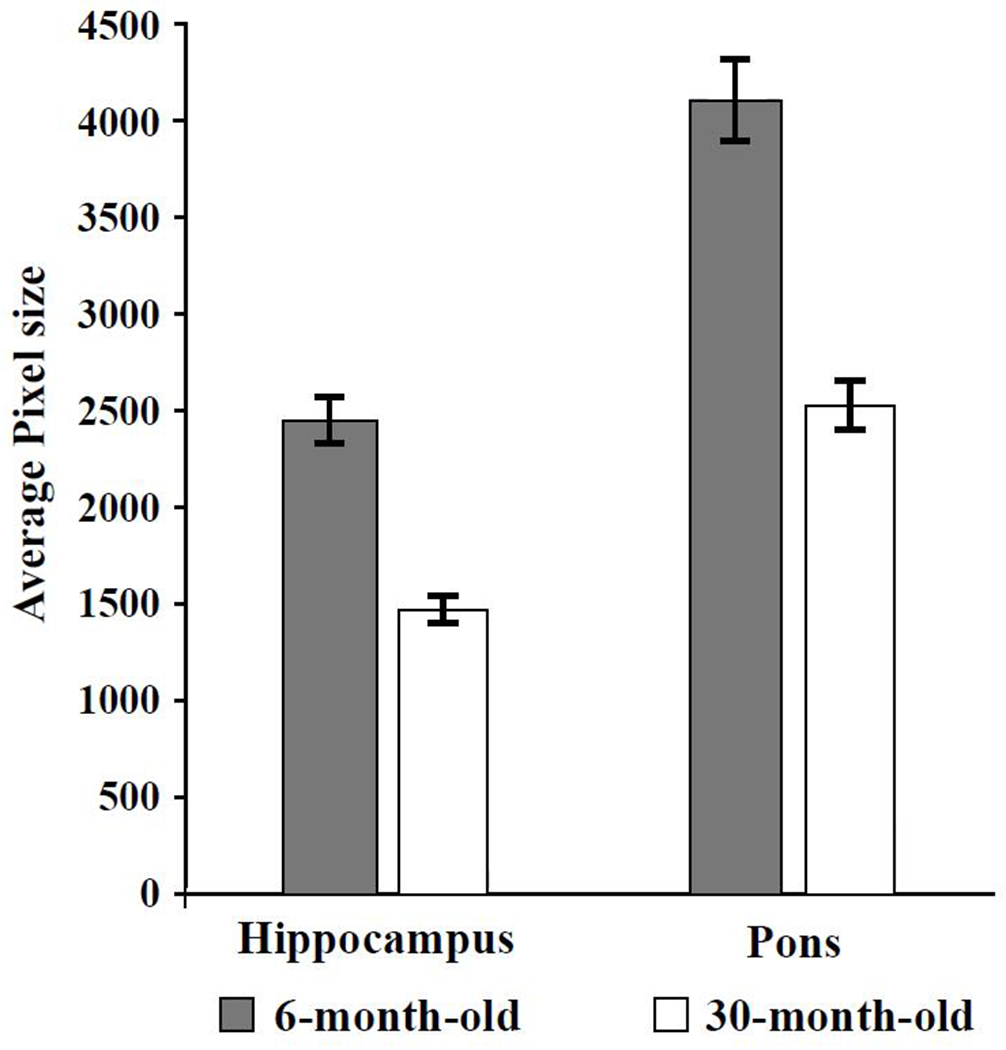
The hippocampal and pons areas were smaller in 30-month-old CB6F1 mice compared to 6-month-old CB6F1 mice. Pixel areas were standardized to tibial length for age comparison, with a *p*-value of 0.01 for both the hippocampus and the pons. N = 5 per age group.

**Figure 3.**
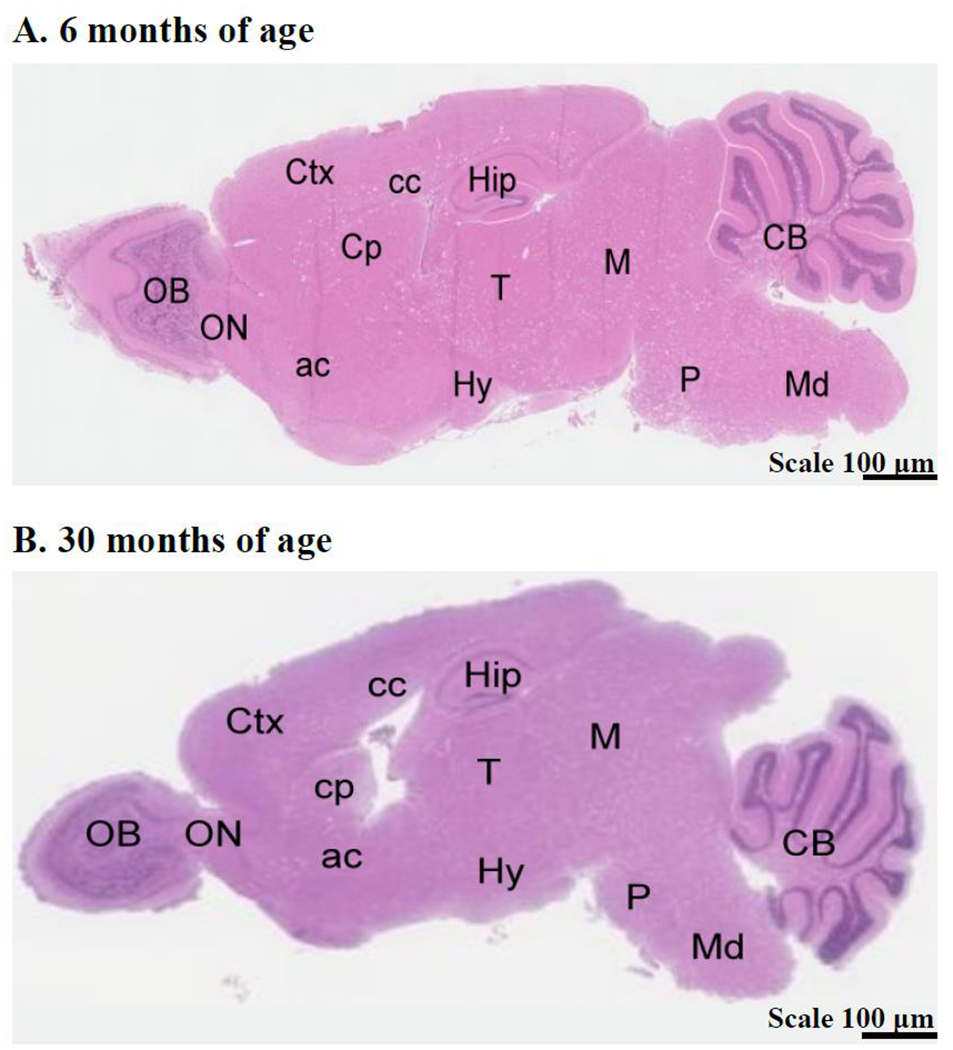
Hematoxylin and eosin-stained sagittal brain sections of a 6-month-old and a 30-month-old CB6F1 mouse show the regions measured in this study. Abbreviations: Ctx = cerebral cortex, Cp = caudate putamen, cc = corpus callosum, Hip = hippocampus, M = midbrain, CB = cerebellum, OB = olfactory bulb, ON = olfactory nucleus, ac = anterior commissure, T = thalamus, Hy = hypothalamus, P = pons, Md = midbrain

## Data Availability

Not applicable.
